# Pre-infection cerebral cortex structure predicts murine sepsis outcome

**DOI:** 10.1371/journal.pone.0330947

**Published:** 2025-09-17

**Authors:** Robert M. Gallant, Shahinur Alam, Krishnan Venkataraman, Benjamin S. McKenna, Khaled Khairy, Janelle S. Ayres

**Affiliations:** 1 Molecular and Systems Physiology Lab, Salk Institute for Biological Studies, La Jolla, California, United States of America; 2 Division of Biological Sciences, University of California, San Diego, La Jolla, California, United States of America; 3 NOMIS Center for Immunobiology and Microbial Pathogenesis, Salk Institute for Biological Studies, La Jolla, California, United States of America; 4 Gene Expression Lab, Salk Institute for Biological Studies, La Jolla, California, United States of America; 5 Center for Bioimage Informatics, St. Jude Children’s Research Hospital, Memphis, Tennessee, United States of America; 6 Pacific Treatment and Research Center, Department of Psychiatry, University of California, San Diego, San Diego, California, United States of America; 7 Department of Developmental Neurobiology, St. Jude Children’s Research Hospital, Memphis, Tennessee, United States of America; 8 Howard Hughes Medical Institute, The Salk Institute for Biological Studies, La Jolla, California, United States of America; Gifu University School of Medicine Graduate School of Medicine: Gifu Daigaku Igakubu Daigakuin Igakukei Kenkyuka, JAPAN

## Abstract

Sepsis is a life-threatening condition caused by an inappropriate host response to an infection that can lead to multi-organ failure and death. Predictive markers that can inform the trajectory and outcome for a septic patient are necessary to inform treatment and increase the likelihood for patient survival. Here, by leveraging the concept of Lethal Dose 50 (LD50), which is the dose of a pathogen that will kill 50% of a genetically identical host population, we tested the hypothesis that variations in brain structure can be readily used to predict trajectory and outcome in a murine model of sepsis. We found that one week prior to infection, mice that were fated to survive exhibited greater cortical volume, solidity, and thickness compared to those who would succumb to the LD50 challenge, and these metrics were sufficient to train multiple predictive models with 75% to 94% accuracy. Our work reveals a readily measurable non-genetic marker for predicting sepsis prognosis in mice and demonstrates the potential for using pre-infection high-resolution structural brain scans to predict infection outcomes in humans.

## Introduction

For many pathogens, virulence (the ability to cause disease) exists on a spectrum. While infection in one host can lead to disease and death, another host infected with the same pathogen can be entirely asymptomatic [1–5]. There is an urgent need to not only understand the pathogen and host factors that contribute to this variation, but also to identify markers that can predict the disease trajectory a host will follow upon infection and the eventual outcome. Such predictive markers have the potential to inform intervention strategies for infection settings where timely treatment is critical to increase the likelihood for patient survival. Sepsis is defined as a life-threatening organ dysfunction caused by a dysregulated host response to infection that progresses rapidly in patients, and timely interventions are essential [6–8]. Several groups have developed and refined machine learning algorithms based on vital signs and circulating biomarkers, such as blood pressure, heart rate and lactate levels upon hospitalization, that can predict whether a hospitalized individual suffering from an infection will progress to sepsis [9–11]. While these algorithms can exhibit impressive sensitivity and specificity, they are limited to predicting sepsis only up to 12–24 hours before symptom onset in hospitalized individuals. We sought to identify quantifiable markers that exist in uninfected hosts and that can reliably predict sepsis trajectory and outcome.

Brain structure and function can vary significantly in animals and humans independent of genetics and environment [12–16]. This heterogeneity is thought to be driven by the inherent stochasticity involved in neurodevelopment [17]. While the genome encodes the genetic plans to make an organism, there can be stochastic differences in the timing and location of the signals involved in the developmental plan of the brain, such as axon guidance and synapse formation cues that can lead to structural and functionally relevant differences [18]. Structural differences in the brain have been shown to have predictive power for neurodegenerative disorders including Alzheimer’s and Huntington’s diseases [19,20]. Several studies suggest the earliest abnormality that leads to organ dysfunction during sepsis arises in the brain [21–23], thus it is perhaps plausible that non-genetic differences in brain structure can predispose individuals to follow different health trajectories after systemic microbial challenge. In the current study, we tested the hypothesis that variations in brain structure can also be used to predict disease trajectory and outcome in septic hosts.

Recent advancements in machine learning, especially deep learning [24], have increased the accuracy of object detection [25], image classification [26,27], and medical image segmentation [28,29]. These successes in medical image segmentation have facilitated breakthroughs in automated morphometric analysis, which improve predictive models’ robustness and diagnostic accuracy [30,31]. In the current study, using a mouse model of polymicrobial sepsis and deep learning, we demonstrate that pre-infection brain structure differences of the cerebral cortex correlate with sepsis outcome and can be successfully used to train a machine learning algorithm to predict sepsis prognosis. Our study highlights the potential for using pre-infection high-resolution structural brain scans to predict infection outcomes in a murine polymicrobial sepsis model, which may inform efforts to predict sepsis outcomes in humans.

## Materials and methods

### Ethics statement

All animal experiments were done in accordance with The University of California San Diego Animal Care and Use Committee number #S17212.

### Mice

Female mice of 10–12 weeks of age purchased from Jackson Labs were used for the studies described. Animals were acclimated in our facility for at least a week prior to experimentation. Mice were specific pathogen-free, maintained under a 12-hour light/dark cycle, and given standard chow diet *ad libitum* prior to infection. All animal experiments were done in accordance with The University of California San Diego Animal Care and Use Committee number #S17212 including humane endpoints to minimize suffering and distress. All animal handlers were trained in all in vivo protocols used for this study and identifying humane endpoints prior to commencing experimentation.

### Image acquisition

In vivo MRI scanning was conducted 1 week pre-infection between 09:00 and 18:00. Experiments were conducted at the UCSD Center for Functional MRI using a Bruker 7.0T/20 cm horizontal magnet with Avance II hardware (Bruker, Billerica, MA, USA). Mice were anesthetized with an isoflurane-oxygen mixture (2.0 vol% with an oxygen flow of 1.2–1.4 l/min) in order to minimize motion throughout the session. Mice were positioned prone in an animal holder with foam pads on each side of the head and front teeth were hooked onto a bite bar. Body temperature was monitored by a rectal thermometer and kept at ~37°C core temperature using warm airflow. A high-resolution anatomical dataset was collected using a RARE (Rapid Acquisition with Relaxation Enhancement) pulse sequence (TR/TE = 9247/35ms, RARE factor = 8, bandwidth = 35,714 Hz, field of view 12.8 mm-x-12.8 mm, matrix 256-x-256, in-plane resolution 0.05 mm-x-0.05 mm, 50 slices 0.3 mm thick, averages = 4, flip angle 85°). Data was acquired using a two-channel local receive coil combined with a 72 mm ID birdcage volume transmitter.

### Bacteria strains used in study

*Escherichia coli* O21:H+ [32]

*Staphylococcus aureus* (ATCC strain 12600)

### Culturing E. coli O21:H+ and S. aureus for mouse infection

*E. coli* O21:H + was incubated on an EMB plate containing Ampicillin sodium salt (1 mg/mL), Vancomycin hydrochloride (0.5 mg/mL), Neomycin sulfate (1 mg/mL), and Metronidazole (1 mg/mL) antibiotics while *S. aureus* was incubated on an LB plate without antibiotics overnight at 37°C to grow single colonies. The next day, a single colony of *E. coli* O21:H + was inoculated into 100mL LB-AVNM media and a single colony of *S. aureus* was inoculated into 5mL LB without antibiotics. Both cultures were shaken overnight at 37°C (250 RPM). The following morning, the OD600 was measured and an inoculum with a 1:1 mixture of the bacteria was prepped with the appropriate amount of bacteria in sterile 1xPBS that was used directly for mouse infections.

### Mouse infection models

Mice were infected intraperitoneally with 2x10^8^ total bacteria in 500uL delivered through a 25G needle between ZT0 and ZT1 (8:00am – 9:00am). Inoculums were serially diluted and plated to confirm the infectious doses. Immediately after infection, mice were transferred to a fresh cage and food was removed for the first 14 hours post-infection to control for any potential variations in the sickness-induced anorexic response. Mice were clinically monitored as described below every two hours post-infection for the first 14 hrs post-infection and then every 24 hrs. We established a grading system to monitor and quantify morbidity to identify when animals reached humane clinical endpoints to alleviate pain and suffering. This system is described below. Mice that reached clinical endpoints were euthanized immediately by carbon dioxide asphyxiation followed by cervical dislocation as a secondary means of euthanasia in accordance with our animal protocol.

### Survival

Mice were clinically monitored as described below every two hours post-infection for the first 14 hours post-infection and then again at 24 and 48 hours. Mice that had to be euthanized because they reached clinical endpoints during the infection, in addition to those that succumb to the infection prior to reaching clinical endpoint, were included in our survival analyses. A score of 1 (moribund) was used as our humane clinical endpoint. Animals were immediately euthanized once they reached clinical endpoint. In our study, 36 mice reached clinical endpoint and were humanely euthanized. 7 animals succumbed to the infection prior to reaching clinical endpoint.

### Rectal temperature

Rectal temperatures were taken every two hours post infection for the first 14 hours, then again at 24 and 48 hours using the Digisense Type J/K/T thermocouple meter.

### Grading system for monitoring morbidity and humane endpoints

We use the following morbidity scale to quantify the morbidity of mice. Infected mice are clinically assessed using this morbidity scale every two hours post-infection. Mice were clinically monitored every two hours for the first 14 hours post-infection and then again at 24 and 28 hours.

5. Normal. Normal exploratory behavior, rearing on hind limbs, and grooming.
4. Mild. Reduced exploratory behavior, rearing on hind limbs, and grooming. Slower and/or less steady gait, but free ambulation throughout the cage.
3. Moderate. Limited voluntary movement. Slow, unsteady gait for >5 seconds.
2. Severe. No voluntary movement, but the mouse can generate slow, unsteady gait for <5 seconds.
1. Moribund. Mouse does not move away from stimulation by researchers and cannot right itself.

Once animals reach a score of “1” they have reached the humane clinical endpoint and are euthanized. Animals were immediately euthanized once they reached clinical endpoint. In our study, 36 mice reached clinical endpoint and were humanely euthanized. 7 animals succumb to the infection prior to reaching clinical endpoint.

### Skull Stripping

We adopted state-of-the-art deep learning-based model, DeepBrainIPP as described in [33], to perform skull Stripping. DeepBrainIPP is a Unet [34] based model consists of an encoder and decoder with Residual unit [32,35]. Initially, we used a pre-trained model to segment the brain from the skull. The pre-trained model overestimated and underestimated the segmentation boundary for some MRI images. S1 Fig demonstrates skull tripping outcomes for a sample volume with pre-trained versus updated/tuned DeepBrainIPP. The possible reasons for over/under estimations are: 1) DeepBrainIPP model was trained on MRI volumes that were acquired with different imaging (geometric and photometric) parameters compared to ours (for example, TR/TE 9247/35ms versus 2,500/42 ms; field of view 25 mm x 25 mm versus 12.8 mm X 12.8 mm). Hence, resolution and contrast of our MRI images may be significantly different than DeepBrainIPP dataset. 2) There might be anatomical disparity since mice were from different age groups (DeepBrainIPP: 2−8 weeks ours: female 10−12 weeks). To improve segmentation performance, we included 12 MRI images (6 from survivor and 6 from non-survivor) with publicly available DeepBrainIPP dataset and retrained the model. The MRI volumes obtained from two data sources were resampled to 0.06 mm x 0.06 mm x 0.48 mm to make the resolution uniform. We applied data augmentation techniques on 12 MRI images and generated 240 volumes to make the model robust against photometric and geometric changes. The data augmentation methods used are horizontal flip, dropout pixels, sharpening, Gaussian smoothing, elastic deformation, piecewise Affine and Affine transformation. We used 80% of the dataset for training and 20% for validation. The model was trained on 7500 MRI volumes of dimension 448 x 448 x 48 on NVIDIA A100 GPUs with a batch size of 1 (limited by available memory) and epochs of 300 for 7 days. The optimal values of hyperparameters (depth of network, initial learning rate, dropout rate, and number of filters in base layer) of DeepBrainIPP network obtained from a comprehensive grid search are 5, 5e-5, 0.10 and 16. The detailed implementation and codebase can be found here: https://github.com/stjude/DeepBrainIPP.

### Image registration

Image registration is a process of finding geometric transformation to align a moving image to a target image/atlas/template. Image registration facilitates segmentations of region-of-interest (ROIs) when the target image is delineated into labeled-mask. In this study, we examined 10 brain structures (External-Capsule, Hippocampus, Cerebellum, Brain-stem, Ventricles, Olfactory-bulb, Cortex, Hypothalamus, Thalamus, Caudate-Putamen). Segmentation of these regions manually would be laborious. The segmentation via image registration is indispensable when manual annotation of a large number of ROIs is exhaustive. However, segmentation based on image registration depends on modalities of moving and target images and preciseness of their alignment. We experimented registration quality of our MRI images with NeAt [36] templates, Allen atlas [37] and DeepBrainIPP template using web-based DeepBrainIPP pipeline. DeepBrainIPP uses ANTs [38] as backbone for the image registration. The Allen atlas and NeAt template were downsampled to 0.05 mm × 0.05 mm × 0.314 and 0.047 mm × 0.047 mm × 0.377 mm respectively to bring spatial resolution close to our MRI images. The transformation models used to register volumes are Rigid followed by Affine followed by Syn [39]. We first optimized the parameters of the transformation models for a volume and then used those parameters to register the rest of the volumes. The Table 1 below shows optimal values of transformation parameters obtained from grid search. We calculated normalized cross-correlation (NCC) to quantify registration and DeepBrainIPP template produced best score (an average NCC of 0.95) compared to NeAt (an average NCC of 0.87) and Allen atlas (an average NCC of 0.75). Moreover, two domain experts manually examined each registered volume to discover possible anomalies. The brain structures from registered volumes were segmented by applying inverse transformation to the template mask. The detailed implementation and codebase can be found here: https://github.com/stjude/DeepBrainIPP.

**Table 1:** Optimal transformation parameter values for image registration.

Transformation Model	Parameter	Parameters Value
Rigid	Gradient Step	0.1
	Metric	Mutual Information
	Shrink factor	8, 4, 2, 1
	Smoothing Sigma	8, 6,2,1
	Number of Bins	32
	Sampling Strategy	Regular (50% sample)
	Number of levels	4
	Number of iterations	1000, 500, 300, 100 (early stop with epsilon of 1e-6 and Patience of 20)
Affine	Gradient Step	0.1
	Metric	Mattes
	Shrink factor	8, 4, 2, 1
	Smoothing Sigma	8, 6, 2, 1
	Number of Bins	32
	Sampling Strategy	Regular (50% sample)
	Number of levels	4
	Number of iterations	1000, 500, 300,100 (early stop with epsilon of 1e-6 and Patience of 20)
Syn	Gradient Step	0.1
	Metric	Cross Correlation (CC)
	Shrink factor	8, 4, 2, 1
	Smoothing Sigma	8, 6, 2, 1
	Number of Bins	4
	Sampling Strategy	Regular (50% sample)
	Number of levels	4
	Number of iterations	400, 200, 100, 50 (early stop with epsilon of 1e-6 and Patience of 20)

### Morphological measurements

The morphological measurements of volume, solidity, and length of major and minor axes were measured using scikit-image (https://scikit-image.org/) library.

### Cortical thickness

Cortical thickness estimates were generated on the pial surface of cortex masks using the following approach [40]. The outer pial boundary and inner white matter boundaries were defined by a series of steps dilating and eroding the cortex masks and the whole brain masks. Values of a potential function ψ defined within these boundaries were then calculated using a Jacobi-type iterative algorithm to solve Laplace’s equation, constructed as follows:

$\begin{array}{cc} \Delta \psi =\frac{\partial^{2}}{{\partial x}^{2}}\psi +\frac{\partial^{2}}{{\partial y}^{2}}\psi +\frac{\partial^{2}}{{\partial z}^{2}}\psi =0 & on\;\Omega \\ \psi =\psi_{0} & on\;\partial \Omega \end{array}$

where Ω corresponds to the interior of the cortex region, and ∂Ω is its boundary. $\psi_{0}$ corresponds to the following boundary condition: the value of ψ for each voxel on the outer pial boundary was set to 0 and the value of ψ for each voxel on the inner white matter boundary was set to 1. Δψ was then calculated using a first-order approximation at each voxel, and paths were traced according to these gradients from each point on the outer pial boundary to the inner white matter boundary. The length of each path was calculated to determine the cortical thickness under each point on the pial boundary. Average cortical thicknesses within each z-slice and across the entire cortex were calculated then statistically compared. Since cortical thickness was calculated on scans in their native spaces, z-slices were compared by registering the first slice with cortical tissue as the origin for each mouse. Cortical thickness maps were generated using ParaView 5.11.2. ChatGPT 4.0 (OpenAI) was used to help write and troubleshoot the code for this task. The detailed implementation and codebase can be found here: https://github.com/stjude/Mouse_Survival.

### Statistical analyses

A Two-Way ANOVA with Sidak multiple comparisons test was conducted within each brain region on extracted volume data as well as on z-slice averaged cortical thicknesses. Cortical volume, solidity, thickness, major and minor axis lengths were tested for normality. Parameters with normally distributed data were compared with a two tailed t-test, non-normal data was compared with a Mann-Whitney U-test. These tests were all conducted in Prism GraphPad 8.4.3.

### Predictive models

The predictive model was built on the morphological measurements obtained from the segmented brain structures. There are several algorithms that can be utilized to build a predictive model considering the size of our dataset, underlying patterns, and assumptions. We experimented with Support Vector Machine (SVM), Random Forest, Logistic Regression, Neural Network and TabNet [41]. The models were trained on 80% of our dataset and 20% were used for validation. The training and test samples were selected randomly and equally to ensure the model learns sufficient representation from both survivors and non-survivors. The network consists of an expansion pathway and compression pathway with a depth/level of 4. The expansion pathway projects the input data in higher dimension (number of base filters 16) to increase separability, whereas, the compression pathway reduces high-level abstract gradually and performs class-wise assignment using Sigmoid activation. Each block of expansion and compression pathways contains a Dense layer, BatchNormalization layer, PRelu activation layer and Dropout Layer. BatchNormalization helps to prevent covariant shift, ease weight initialization and accelerate training. Dropout is included to prevent overfitting since our dataset is very small. The number of filters is increased/decreased by a factor of 4 in each level of expansion/compression pathway. The network was trained using Stochastic Gradient Descent (SGD) optimizer for 1500 epochs (with an early stopping if validation score does not improve) with a batch size of 32, learning rate of 0.01 and momentum of 0.9. We used 1-F1 score as a loss function and accuracy as a metric. The detailed implementation and codebase can be found here: https://github.com/stjude/Mouse_Survival

## Results

### Study design to determine the predictive value of pre-existing variations in brain structure for sepsis outcome

**Fig 1:**
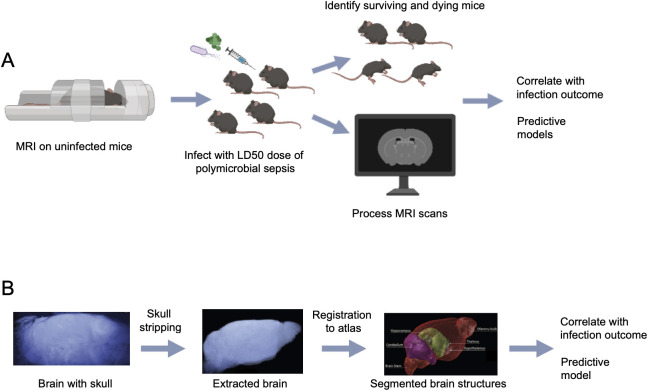
Experimental approach and MRI processing pipeline. A: Schematic of experimental approach. T2-weighted MRIs of brains were collected from C57BL/6J mice. One week later these mice were challenged with an LD50 of polymicrobial sepsis and infection outcomes were tracked. Brain scans and sepsis outcomes were then analyzed to determine if pre-existing differences can predict survival. B: Schematic of image processing pipeline. Scans were skull stripped, registered to the DeepBrainIPP atlas, and segmented into brain structures. These structures were then investigated for morphological differences that could train a model to predict sepsis outcome.

For our studies, we leveraged the phenomenon of lethal dose 50 (LD50), which describes the dose of a pathogen that will kill 50% of a genetically identical host population while the other half survives. We elected to use a polymicrobial sepsis model consisting of an intraperitoneal injection of a 1:1 mixture of *Escherichia coli* and *Staphylococcus aureus*, two of the most commonly identified gram-negative and gram-positive microbes in septic patients [42]. While the kinetics of disease progression with this model is faster than that what is observed in human sepsis, we previously established this model causes multi-organ dysfunction and damage in mice, which is the defining characteristic of sepsis [43,44]. Importantly, it offers both the clinical relevance and generalizability of multiple live microbes, and the consistency of cultured pathogens that is much harder to achieve with other common murine sepsis models such as cecal ligation and puncture (CLP) or fecal slurry injection. A schematic of our experimental approach is shown in Fig 1A (see methods for detailed description). Briefly, across two independent experiments, we employed *in vivo* T2-weighted magnetic resonance imaging (MRI) and collected brain scans of 80 C57Bl/6J mice. One week post imaging, we intraperitoneally infected mice with the LD50 dose for our polymicrobial sepsis model which consists of a 1:1 mixture of *Escherichia coli* and *Staphylococcus aureus*. We then processed the MRI scans with a previously established automated end-to-end pipeline called DeepBrainIPP (deep learning-based brain image processing pipeline) [33] (Fig 1B). Specifically, we skull stripped the scans using a 3D U-Net based model, then registered the scans to the DeepBrainIPP atlas, segmented the brains into 10 major regions, and combined the unsegmented regions into a “rest-of-brain” region (S1 Fig). We extracted several metrics from these segmented regions and conducted statistical analyses to determine whether there were any pre-existing differences in brain structure between survivors and non-survivors before infection. We then determined whether any identified pre-existing differences in brain structure could be leveraged to train a predictive model and compared the success of different models.

**Fig 2:**
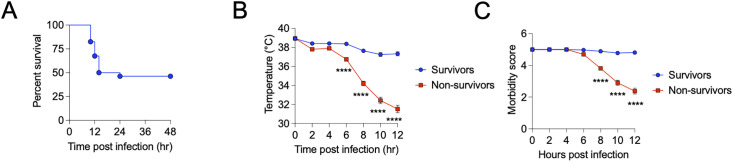
Polymicrobial sepsis LD50 model. A: Survival of C57BL/6J mice intraperitoneally infected with the LD50 dose of a 1:1 mixture of *E. coli* and *S. aureus*. n = 37 survivors and 43 non-survivors, two independent experiments combined. B: Time course of body temperature of C57BL/6J mice intraperitoneally infected with the LD50 dose of a 1:1 mixture of *E. coli* and *S. aureus*. n = 37 survivors and 43 non-survivors, two independent experiments combined. Two-Way ANOVA with Sidak multiple comparisons test. Error bars indicate + /- SEM. C: Time course of morbidity scores of C57BL/6J mice intraperitoneally infected with the LD50 dose of a 1:1 mixture of *E. coli* and *S. aureus*. n = 37 survivors and 43 non-survivors, two independent experiments combined. Two-Way ANOVA with Sidak multiple comparisons test. Error bars indicate + /- SEM.

Of the 80 mice imaged and challenged with polymicrobial sepsis, 37 survived, and 43 were identified as non-survivors by 48 hrs post-infection with a median time to death of 12 hrs (Fig 2A). Mice on the trajectory to death exhibited quantifiable clinical signs of sickness including hypothermia (Fig 2B) and overall morbidity (Fig 2C). For three mice, we were unable to successfully segment the MRIs, most likely due to excessive noise from movement in the scanner (1 survivor and 2 non-survivors). Thus, our final image dataset consisted of MRIs of 36 survivors and 41 non-survivors for our analyses.

### Pre-existing differences in brain structure correlates with sepsis outcome

**Fig 3:**
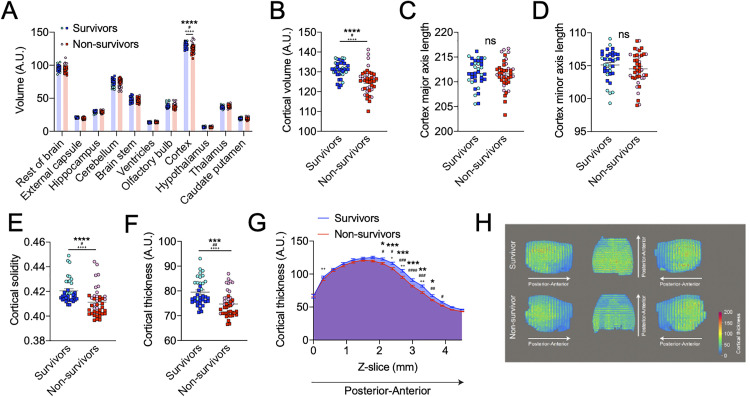
Pre-infection cortical structure varies between mice that survive and succumb to polymicrobial sepsis. A: Comparison of pre-infection brain region volumes. Two-way ANOVA with Sidak multiple comparisons test. n = 36 survivors and 41 non-survivors, two independent experiments combined. Experiment #1 n = 14 survivors and 16 non-survivors, experiment #2 n = 22 survivors and 25 non-survivors. B-F: Pre-infection B: cortical volume, C: major axis length, D: minor axis length, E: solidity and F: thickness. Unpaired t-tests for volume, axis lengths, and thickness, Mann-Whitney test for solidity. n = 36 survivors and 41 non-survivors, two independent experiments combined. Experiment #1 n = 14 survivors and 16 non-survivors, experiment #2 n = 22 survivors and 25 non-survivors. G: Pre-infection cortical thickness across Z-slices. Two-way ANOVA with Sidak multiple comparisons test. Two independent experiments combined. Experiment #1 n = 14 survivors and 16 non-survivors, experiment #2 n = 22 survivors and 25 non-survivors. H: Representative survivor and non-survivor cortical thickness maps. Data summaries represent mean ± SEM. Circle data points represent mice from experiment #1 while square data points represent mice from experiment #2. # symbols indicate statistical significance in experiment #1, + symbols indicate statistical significance in experiment #2, and * symbols indicate significance in both experiments combined. * p < 0.05, ** p < 0.01, *** p < 0.001, **** p < 0.0001.

From our analyses, we found that the survivors on average had 4.7% larger cerebral cortexes compared to non-survivors, with no significant volume differences observed in any other segmented brain region (Fig 3A–B). Given this difference in cortical volume, we next sought to elucidate other cortical structure differences between survivors and non-survivors. While we did not find differences in cortical length along major (anterior to posterior) or minor (left to right) axes (Fig 3C–D), we found increased cortical solidity (solid area divided by gross area) (Fig 3E). Furthermore, we found survivors had on average 6.4% thicker cerebral cortexes compared to non-survivors, and these differences were driven specifically by a thicker band 0.9 mm-2.4 mm anterior to the rear edge of the cortex (cortical thickness calculations are outlined in the methods) (Fig 3F–G). Importantly, these findings held true for each independent experimental repeat (S2 Fig). Representative images of cortical thickness maps from survivors and non-survivors are demonstrated in Fig 3H. Together these data indicate that pre-existing differences in cortical structure correlate with differences in infection outcome in a murine model of polymicrobial sepsis.

### Sepsis outcome can be predicted by differences in cortex structure that exist prior to infection

**Fig 4:**
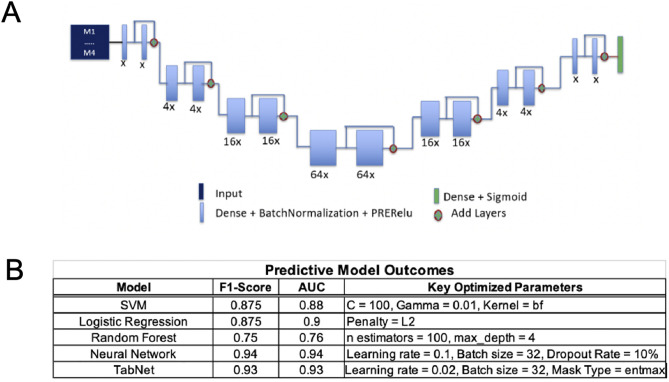
Pre-infection cortical structure successfully trains machine learning models to predict murine polymicrobial sepsis outcome. A: Neural network architecture. B: Predictive model outcomes. See Predictive Models section of Experimental Procedures for more details.

We next determined whether these pre-existing differences in cortical structure can be leveraged to train a predictive model to predict sepsis outcome. We trained five different predictive models with the cortical volume, major axis length, minor axis length, solidity, and thickness from 80% of mice from our dataset, and tested their prediction accuracy on the remaining 20%. We found that a support vector machine, a logistic regression model, Random Forest, a neural network, and TabNet (Fig 4A) all performed with comparable accuracy, 87.5%, 87.5%, 75%, 94%, and 93% respectively (Fig 4B). Together these data indicate that pre-infection cortical structure can be used to train a model to predict sepsis trajectories and outcomes.

## Discussion

In the present study, we demonstrate that the use of predictive algorithms and structural brain scans can extend to predicting infectious disease outcomes. We demonstrate that surviving mice that were challenged with the LD50 dose of a polymicrobial sepsis challenge have larger, thicker cortices. These differences were detectable one week prior to infection by in-vivo T2-weighted MRI scans processed using DeepBrainIPP – an automated end-to-end pipeline that segments scans into major structural regions. We further show that these data could be used to successfully train models to predict sepsis outcome with between 75% and 94% accuracy. While our study identified the cortex as a prognostic marker for sepsis trajectory and outcome, we cannot formally exclude the possibility that structural differences in other regions may also be used to predict sepsis outcome, but were possibly not detectable by our experimental approach and sample size.

Our findings contribute to ongoing efforts to identify prognostic biomarkers for sepsis. Several studies have trained machine learning algorithms to predict sepsis onset or sepsis outcome in hospitalized patients using data such as electronic medical records (EMR), vital signs like blood pressure and heart rate data, and blood work including electrolytes and white blood cell counts [9–11,45]. However, these efforts have a short predictive window of 12–24 hrs prior to septic symptom onset after hospitalization. While our study is in mice, we were able to predict sepsis outcome a full week in advance of infection challenge using structural brain data. Other groups have successfully used structural brain data to predict Alzheimer’s onset in humans up to 3 years in advance [19], and Huntington’s disease up to 5 years in advance [20]. It will be important to determine in future studies how large of a predictive window there is for the utilization of pre-existing differences in brain structure for predicting sepsis outcomes. Furthermore, it will be important to determine whether our findings can be applied to human sepsis as well as other infectious diseases.

While we currently do not know if variations in cortical structure play a functional role in sepsis pathogenesis or defense, it’s tempting to speculate the potential ways in which the cortex may do so. Previous work has demonstrated that LD50 survivors of the polymicrobial sepsis model do not develop systemic organ damage as measured by circulating markers and histopathology [44]. These mice do develop a systemic infection as reflected by high pathogen burdens across their heart, lungs, liver, kidneys, and spleen. Thus, their ability to remain healthy and survive the infection challenge is not due to a heightened resistance response and ability to clear the infection, but rather because they are better able to tolerate the infection [46]. Indeed, transcriptional analyses of the hearts of the polymicrobial sepsis LD50 survivors demonstrated a unique infection-inducible cardiac transcriptome that revealed a FoxO1-dependent disease tolerance mechanism that was necessary to enable mice to survive a polymicrobial systemic challenge [44]. FoxO1 is activated by insulin/IGF-1 signaling, which is primarily regulated by the hypothalamic-pituitary axis [47]. It is interesting to speculate that the LD50 survivors harbor pre-existing differences in brain structure that lead to an induction of insulin/IGF-1 signaling which ultimately results in FoxO1-mediated cardiac protection.

With regard to more specific cortical functions, the cortex is known to integrate signals from visceral organs and then coordinate the autonomic nervous system through relays in the hypothalamus and brainstem, a critical circuit during sepsis [48]. The cortex is also known to be susceptible to damage that occurs in sepsis-associated encephalopathy (SAE) [49] and mice succumbing to the sepsis model cecal ligation and puncture (CLP) demonstrate decreased MRI-measured cortical apparent diffusion coefficients [50]. Therefore, pre-existing differences in cortical thickness may protect or sensitize some mice to neural damage. It is also possible that an external third factor independently influences both cortex development and sepsis survival. For example, feeding is known to influence both cortical structure and sepsis outcome [51,52], perhaps variations in eating between mice within the same cage explain these differences. Regardless, this work and many other previous studies support the conclusion that the earliest abnormality that leads to sepsis-related organ dysfunction may arise in the brain [8,21–23]. Future work is needed to understand a functional relationship between cortex structure and sepsis susceptibility, and whether cortex structure or other brain regions can be used to predict outcome for other types of infections.

This research was conducted solely with female mice to minimize variability associated with sex-based differences in sepsis responses and brain structure. More specifically, females are generally less susceptible to sepsis-induced mortality compared to males, a difference attributed in part to hormonal and immunological factors, both in mice and humans [53–55]. Additionally, females tend to have larger cortical volumes than males, again both in mice and humans [56,57]. These conserved differences indicate that the observed relationship between cortical volume and infection survival may differ by sex. Future studies incorporating male mice will be important to determine the generalizability of these findings across sexes.

More generally, our findings underscore a crucial caveat in laboratory research: inbred mice, traditionally considered uniform subjects, exhibit significant pre-existing differences that may substantially influence experimental results. This study centered on variations in brain structure and its relationship with infection outcome; however other factors that vary within a cage may also play pivotal roles in shaping experimental outcomes. Consequently, variations observed within experimental groups should not be viewed as entirely stochastic noise and nuisances, but rather as potentially biologically relevant differences and opportunities. Additionally, the failure of a preclinical intervention to uniformly affect individuals may be due to critical non-genetic and yet-to-be identified biological variations among subjects. Our insights highlight the importance of researchers identifying and incorporating potential covariates that could account for observed variability in outcomes, even in seemingly homogenous groups and isogenic experiments.

## Supporting information

S1 Fig Skull stripping outcomes for a sample volume. Pre-trained model (left). overestimated flocculus whereas updated/fine-tuned model (right) produced more precise boundary.
(TIF)

S2 Fig Data from Figure 3 split into individual experimental repeats. A-C: Experiment #1, MRI scans collected 4/6/21, 4/7/21, 4/8/21, and 4/9/21, infection conducted 4/15/21. N = 14 survivors and 16 non-survivors. D-F: Experiment #2, MRI scans collected 5/10/21, 5/11/21, 5/12/21, 5/13/21, and 5/14/21, infection conducted 5/18/21. n = 22 survivors and 25 non-survivors. Blue data-points are survivors, red data-points are non-survivors. Same statistical tests as in Figure 3. Data summaries represent mean ± SEM * p < 0.05, ** p < 0.01, *** p < 0.001, **** p < 0.0001.
(TIF)

## References

[1] Furuya-Kanamori L, Cox M, Milinovich GJ, Magalhaes RJS, Mackay IM, Yakob L. Heterogeneous and Dynamic Prevalence of Asymptomatic Influenza Virus Infections. Emerg Infect Dis. 2016;22(6):1052–6. doi: 10.3201/eid2206.151080 27191967 PMC4880086

[2] Ganguly S, Saha P, Guha SK, Biswas A, Das S, Kundu PK, et al. High prevalence of asymptomatic malaria in a tribal population in eastern India. J Clin Microbiol. 2013;51(5):1439–44. doi: 10.1128/JCM.03437-12 23426929 PMC3647920

[3] Gao Z, Xu Y, Sun C, Wang X, Guo Y, Qiu S. A systematic review of asymptomatic infections with COVID-19. Journal of Microbiology, Immunology and Infection. 2021;54(1):12–6. 10.1016/j.jmii.2020.05.001 PMC7227597 32425996

[4] Marineli F, Tsoucalas G, Karamanou M, Androutsos G. Mary Mallon (1869-1938) and the history of typhoid fever. Ann Gastroenterol. 2013;26(2):132–4. 24714738 PMC3959940

[5] Riggs MM, Sethi AK, Zabarsky TF, Eckstein EC, Jump RLP, Donskey CJ. Asymptomatic carriers are a potential source for transmission of epidemic and nonepidemic Clostridium difficile strains among long-term care facility residents. Clin Infect Dis. 2007;45(8):992–8. doi: 10.1086/521854 17879913

[6] Rhodes A, Evans LE, Alhazzani W, Levy MM, Antonelli M, Ferrer R, et al. Surviving Sepsis Campaign: International Guidelines for Management of Sepsis and Septic Shock: 2016. Intensive Care Med. 2017;43(3):304–77. doi: 10.1007/s00134-017-4683-6 28101605

[7] Singer M, Deutschman CS, Seymour CW, Shankar-Hari M, Annane D, Bauer M. The Third International Consensus Definitions for Sepsis and Septic Shock (Sepsis-3). JAMA. 2016;315(8):801. 26903338 10.1001/jama.2016.0287 PMC4968574

[8] Deutschman CS, Tracey KJ. Sepsis: Current Dogma and New Perspectives. Immunity. 2014;40(4):463–75. 24745331 10.1016/j.immuni.2014.04.001

[9] Giannini HM, Ginestra JC, Chivers C, Draugelis M, Hanish A, Schweickert WD, et al. A Machine Learning Algorithm to Predict Severe Sepsis and Septic Shock: Development, Implementation, and Impact on Clinical Practice. Crit Care Med. 2019;47(11):1485–92. doi: 10.1097/CCM.0000000000003891 31389839 PMC8635476

[10] Henry KE, Hager DN, Pronovost PJ, Saria S. A targeted real-time early warning score (TREWScore) for septic shock. Sci Transl Med. 2015;7(299):299ra122. doi: 10.1126/scitranslmed.aab3719 26246167

[11] Nemati S, Holder A, Razmi F, Stanley MD, Clifford GD, Buchman TG. An Interpretable Machine Learning Model for Accurate Prediction of Sepsis in the ICU. Crit Care Med. 2018;46(4):547–53. doi: 10.1097/CCM.0000000000002936 29286945 PMC5851825

[12] Bouchard TJ Jr, Lykken DT, McGue M, Segal NL, Tellegen A. Sources of human psychological differences: the Minnesota Study of Twins Reared Apart. Science. 1990;250(4978):223–8. doi: 10.1126/science.2218526 2218526

[13] Jansen AG, Mous SE, White T, Posthuma D, Polderman TJC. What twin studies tell us about the heritability of brain development, morphology, and function: a review. Neuropsychol Rev. 2015;25(1):27–46. doi: 10.1007/s11065-015-9278-9 25672928 PMC4412550

[14] Loos M, Koopmans B, Aarts E, Maroteaux G, van der Sluis S, Verhage M. Within-strain variation in behavior differs consistently between common inbred strains of mice. Mammalian Genome. 2015;26(7–8):348–54. 26123533 10.1007/s00335-015-9578-7

[15] Power RA, Pluess M. Heritability estimates of the Big Five personality traits based on common genetic variants. Transl Psychiatry. 2015;5(7):e604–e604. 10.1038/tp.2015.96 PMC5068715 26171985

[16] Scholz J, LaLiberté C, van Eede M, Lerch JP, Henkelman M. Variability of brain anatomy for three common mouse strains. Neuroimage. 2016;142:656–62. doi: 10.1016/j.neuroimage.2016.03.069 27046115

[17] White TJH. Brain Development and Stochastic Processes During Prenatal and Early Life: You Can’t Lose It if You’ve Never Had It; But It’s Better to Have It and Lose It, Than Never to Have Had It at All. Journal of the American Academy of Child & Adolescent Psychiatry. 2019 Nov;58(11):1042–50. 31327672 10.1016/j.jaac.2019.02.010

[18] Mitchell KJ. Innate – How the wiring of our brains shapes who we are. Princeton University Press. 2018.

[19] Killiany RJ, Gomez-Isla T, Moss M, Kikinis R, Sandor T, Jolesz F, et al. Use of structural magnetic resonance imaging to predict who will get Alzheimer’s disease. Ann Neurol. 2000;47(4):430–9. 10762153

[20] Mason SL, Daws RE, Soreq E, Johnson EB, Scahill RI, Tabrizi SJ, et al. Predicting clinical diagnosis in Huntington’s disease: An imaging polymarker. Ann Neurol. 2018;83(3):532–43. doi: 10.1002/ana.25171 29405351 PMC5900832

[21] Deutschman CS, Raj NR, McGuire EO, Kelz MB. Orexinergic activity modulates altered vital signs and pituitary hormone secretion in experimental sepsis. Crit Care Med. 2013;41(11):e368-75. doi: 10.1097/CCM.0b013e31828e9843 24105451 PMC6880745

[22] Kressel AM, Tsaava T, Levine YA, Chang EH, Addorisio ME, Chang Q. Identification of a brainstem locus that inhibits tumor necrosis factor. Proc Natl Acad Sci USA. 2020;117(47):29803–10. 33168718 10.1073/pnas.2008213117 PMC7703602

[23] Zaghloul N, Addorisio ME, Silverman HA, Patel HL, Valdés-Ferrer SI, Ayasolla KR. Forebrain cholinergic dysfunction and systemic and brain inflammation in murine sepsis survivors. Front Immunol. 2017;8:1673. 29326685 10.3389/fimmu.2017.01673 PMC5736570

[24] LeCun Y, Bengio Y, Hinton G. Deep learning. Nature. 2015;521(7553):436–44. doi: 10.1038/nature14539 26017442

[25] Anam AI, Alam S, Yeasin M. Expression. In: Proceedings of the 2014 ACM International Joint Conference on Pervasive and Ubiquitous Computing: Adjunct Publication, 2014. 211–4. doi: 10.1145/2638728.2638738

[26] Alam S, Mahmud S, Yeasin M. An Assistive Solution to Assess Incoming Threats for Homes. 2020;.

[27] Krizhevsky A, Sutskever I, Hinton GE. ImageNet classification with deep convolutional neural networks. Commun ACM. 2017;60(6):84–90.

[28] Chi W, Ma L, Wu J, Chen M, Lu W, Gu X. Deep learning-based medical image segmentation with limited labels. Phys Med Biol. 2020;65(23):235001. 10.1088/1361-6560/abc363 PMC8058113 33086205

[29] Wang EK, Chen CM, Hassan MM, Almogren A. A deep learning based medical image segmentation technique in Internet-of-Medical-Things domain. Future Generation Computer Systems. 2020;108:135–44.

[30] Esteva A, Robicquet A, Ramsundar B, Kuleshov V, DePristo M, Chou K, et al. A guide to deep learning in healthcare. Nat Med. 2019;25(1):24–9. doi: 10.1038/s41591-018-0316-z 30617335

[31] Schmitt JE, DeBevits JJ, Roalf DR, Ruparel K, Gallagher RS, Gur RC, et al. A Comprehensive Analysis of Cerebellar Volumes in the 22q11.2 Deletion Syndrome. Biol Psychiatry Cogn Neurosci Neuroimaging. 2023;8(1):79–90. doi: 10.1016/j.bpsc.2021.11.008 34848384 PMC9162086

[32] Ayres JS, Trinidad NJ, Vance RE. Lethal inflammasome activation by a multidrug-resistant pathobiont upon antibiotic disruption of the microbiota. Nat Med. 2012;18(5):799–806. doi: 10.1038/nm.2729 22522562 PMC3472005

[33] Alam S, Eom T-Y, Steinberg J, Ackerman D, Schmitt JE, Akers WJ, et al. An End-To-End Pipeline for Fully Automatic Morphological Quantification of Mouse Brain Structures From MRI Imagery. Front Bioinform. 2022;2:865443. doi: 10.3389/fbinf.2022.865443 36304320 PMC9580949

[34] Navab N, Hornegger J, Wells WM, Frangi AF. Medical Image Computing and Computer-Assisted Intervention – MICCAI 2015. Springer International Publishing. 2015. doi: 10.1007/978-3-319-24574-4

[35] Leibe B, Matas J, Sebe N, Welling M. Computer Vision – ECCV 2016. Springer International Publishing. 2016. doi: 10.1007/978-3-319-46493-0

[36] Ma D, Cardoso MJ, Modat M, Powell N, Wells J, Holmes H, et al. Automatic structural parcellation of mouse brain MRI using multi-atlas label fusion. PLoS One. 2014;9(1):e86576. doi: 10.1371/journal.pone.0086576 24475148 PMC3903537

[37] Wang Q, Ding S-L, Li Y, Royall J, Feng D, Lesnar P, et al. The Allen Mouse Brain Common Coordinate Framework: A 3D Reference Atlas. Cell. 2020;181(4):936-953.e20. doi: 10.1016/j.cell.2020.04.007 32386544 PMC8152789

[38] Avants BB, Tustison N, Johnson H. Advanced Normalization Tools (ANTS). Insight J. 2009;2(365):1–35.

[39] Avants BB, Epstein CL, Grossman M, Gee JC. Symmetric diffeomorphic image registration with cross-correlation: evaluating automated labeling of elderly and neurodegenerative brain. Med Image Anal. 2008;12(1):26–41. doi: 10.1016/j.media.2007.06.004 17659998 PMC2276735

[40] Jones SE, Buchbinder BR, Aharon I. Three-dimensional mapping of cortical thickness using Laplace’s equation. Hum Brain Mapp. 2000;11(1):12–32. doi: 10.1002/1097-0193(200009)11:1<12::aid-hbm20>3.0.co;2-k 10997850 PMC6872107

[41] Arik SO, Pfister T. TabNet: Attentive Interpretable Tabular Learning. AAAI. 2021;35(8):6679–87.

[42] Tabah A, Koulenti D, Laupland K, Misset B, Valles J, Bruzzi de Carvalho F, et al. Characteristics and determinants of outcome of hospital-acquired bloodstream infections in intensive care units: the EUROBACT International Cohort Study. Intensive Care Med. 2012;38(12):1930–45. doi: 10.1007/s00134-012-2695-9 23011531

[43] Gallant RM, Sanchez KK, Joulia E, Snyder JM, Metallo CM, Ayres JS. Fluoxetine promotes IL-10-dependent metabolic defenses to protect from sepsis-induced lethality. Sci Adv. 2025;11(7):eadu4034. doi: 10.1126/sciadv.adu4034 39951524 PMC11827869

[44] Sanchez KK, McCarville JL, Stengel SJ, Snyder JM, Williams AE, Ayres JS. Age-dependent roles of cardiac remodeling in sepsis defense and pathogenesis. bioRxiv. 2023;:2023.03.14.532695. doi: 10.1101/2023.03.14.532695 36993409 PMC10055033

[45] Islam MM, Nasrin T, Walther BA, Wu C-C, Yang H-C, Li Y-C. Prediction of sepsis patients using machine learning approach: A meta-analysis. Comput Methods Programs Biomed. 2019;170:1–9. doi: 10.1016/j.cmpb.2018.12.027 30712598

[46] Ayres JS, Schneider DS. Tolerance of Infections. Annual Review of Immunology. 2012;30(1):271–94. 10.1146/annurev-immunol-020711-075030 22224770

[47] Xing Y q i, Li A, Yang Y, Li X xia, Zhang L nan, Guo H cai. The regulation of FOXO1 and its role in disease progression. Life Sciences. 2018;193:124–31. 29158051 10.1016/j.lfs.2017.11.030

[48] Saper CB. Hypothalamic connections with the cerebral cortex. Prog Brain Res. 2000;126:39–48. doi: 10.1016/S0079-6123(00)26005-6 11105638

[49] Manabe T, Heneka MT. Cerebral dysfunctions caused by sepsis during ageing. Nat Rev Immunol. 2022;22(7):444–58. doi: 10.1038/s41577-021-00643-7 34764472 PMC8582341

[50] Bozza FA, Garteiser P, Oliveira MF, Doblas S, Cranford R, Saunders D. Sepsis-Associated Encephalopathy: A Magnetic Resonance Imaging and Spectroscopy Study. J Cereb Blood Flow Metab. 2010;30(2):440–8. 19844239 10.1038/jcbfm.2009.215 PMC2949132

[51] Liu Z, Neuringer M, Erdman JW Jr, Kuchan MJ, Renner L, Johnson EE, et al. The effects of breastfeeding versus formula-feeding on cerebral cortex maturation in infant rhesus macaques. Neuroimage. 2019;184:372–85. doi: 10.1016/j.neuroimage.2018.09.015 30201462 PMC6230484

[52] Starr ME, Steele AM, Cohen DA, Saito H. Short-term dietary restriction rescues mice from lethal abdominal sepsis and endotoxemia and reduces the inflammatory/coagulant potential of adipose tissue. Critical Care Medicine. 2016;44(7):e509-19. 10.1097/CCM.0000000000001475 PMC4896861 26646465

[53] Kadioglu A, Cuppone AM, Trappetti C, List T, Spreafico A, Pozzi G, et al. Sex-based differences in susceptibility to respiratory and systemic pneumococcal disease in mice. J Infect Dis. 2011;204(12):1971–9. doi: 10.1093/infdis/jir657 22021621

[54] Angele MK, Pratschke S, Hubbard WJ, Chaudry IH. Gender differences in sepsis: cardiovascular and immunological aspects. Virulence. 2014;5(1):12–9. doi: 10.4161/viru.26982 24193307 PMC3916365

[55] Lakbar I, Einav S, Lalevée N, Martin-Loeches I, Pastene B, Leone M. Interactions between Gender and Sepsis-Implications for the Future. Microorganisms. 2023;11(3):746. doi: 10.3390/microorganisms11030746 36985319 PMC10058943

[56] Liu S, Seidlitz J, Blumenthal JD, Clasen LS, Raznahan A. Integrative structural, functional, and transcriptomic analyses of sex-biased brain organization in humans. Proc Natl Acad Sci USA. 2020;117(31):18788–98. 32690678 10.1073/pnas.1919091117 PMC7414084

[57] Guma E, Beauchamp A, Liu S, Levitis E, Ellegood J, Pham L, et al. Comparative neuroimaging of sex differences in human and mouse brain anatomy. Elife. 2024;13:RP92200. doi: 10.7554/eLife.92200 38488854 PMC10942785

