# Supplementary figures and images for: Pre-infection cerebral cortex structure predicts murine sepsis outcome
Source: PLoS One. 2025 Sep 17;20(9):e0330947. doi: 10.1371/journal.pone.0330947 (PMC12443313; doi:10.1371/journal.pone.0330947)

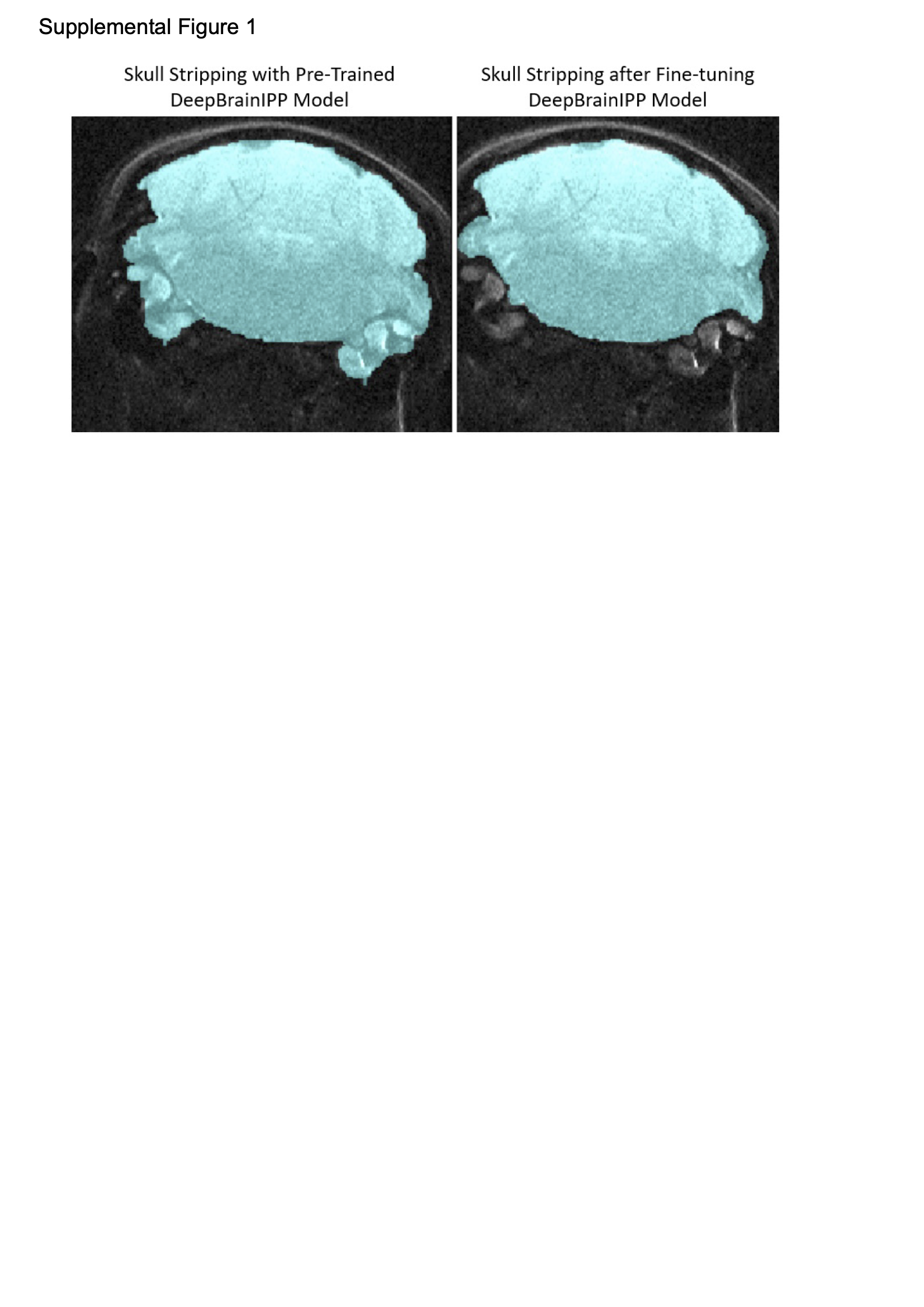

Supplement: S1 Fig — overestimated flocculus whereas updated/fine-tuned model (right) produced more precise boundary. (TIF) [file pone.0330947.s001.tif]

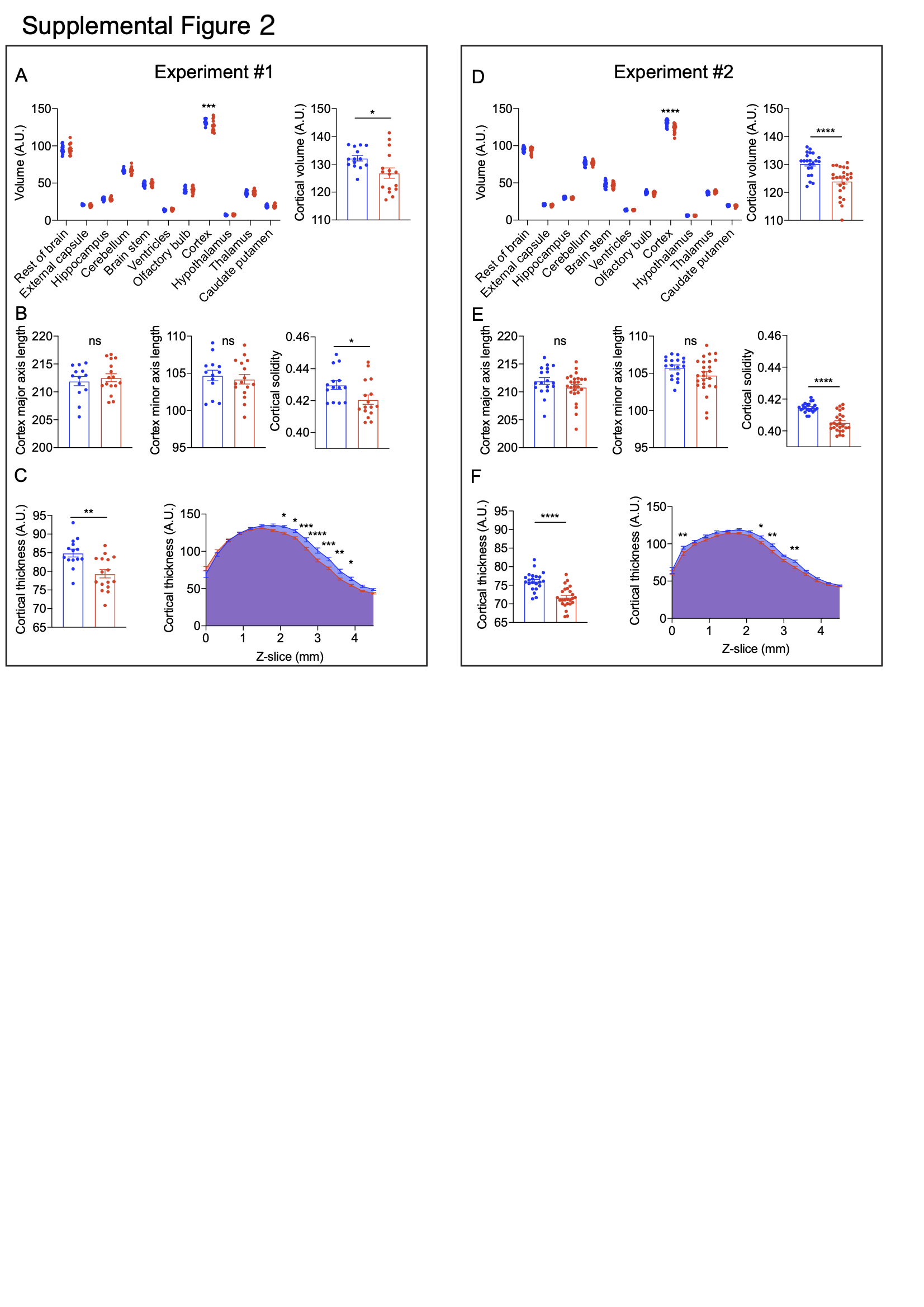

Supplement: S2 Fig — A-C: Experiment #1, MRI scans collected 4/6/21, 4/7/21, 4/8/21, and 4/9/21, infection conducted 4/15/21. N = 14 survivors and 16 non-survivors. D-F: Experiment #2, MRI scans collected 5/10/21, 5/11/21, 5/12/21, 5/13/21, and 5/14/21, infection conducted 5/18/21. n = 22 survivors and 25 non-survivors. Blue data-points are survivors, red data-points are non-survivors. Same statistical tests as in Figure 3. Data summaries represent mean ± SEM * p < 0.05, ** p < 0.01, *** p < 0.001, **** p < 0.0001. (TIF) [file pone.0330947.s002.tif]
